# The Malignant Pleural Effusion as a Model to Investigate Intratumoral Heterogeneity in Lung Cancer

**DOI:** 10.1371/journal.pone.0005884

**Published:** 2009-06-12

**Authors:** Saroj K. Basak, Mysore S. Veena, Scott Oh, Ge Huang, Eri Srivatsan, Min Huang, Sherven Sharma, Raj K. Batra

**Affiliations:** 1 Wadsworth Stem Cell Institute, Veterans Affairs Greater Los Angeles Healthcare System (VAGLAHS*), Los Angeles, California, United States of America; 2 The Jonsson Comprehensive Cancer Center at the Geffen School of Medicine, University of California Los Angeles, Los Angeles, California, United States of America; Emory University, United States of America

## Abstract

Malignant Pleural Effusions (MPE) may be useful as a model to study hierarchical progression of cancer and/or intratumoral heterogeneity. To strengthen the rationale for developing the MPE-model for these purposes, we set out to find evidence for the presence of cancer stem cells (CSC) in MPE and demonstrate an ability to sustain intratumoral heterogeneity in MPE-primary cultures. Our studies show that *candidate* lung CSC-expression signatures (PTEN, OCT4, hTERT, Bmi1, EZH2 and SUZ12) are evident in cell pellets isolated from MPE, and MPE-cytopathology also labels *candidate*-CSC (CD44, cMET, MDR-1, ALDH) subpopulations. Moreover, in primary cultures that use MPE as the source of both tumor cells and the tumor microenvironment (TME), *candidate* CSC are maintained over time. This allows us to live-sort *candidate* CSC-fractions from the MPE-tumor mix on the basis of surface markers (CD44, c-MET, uPAR, MDR-1) or differences in xenobiotic metabolism (ALDH). Thus, MPE-primary cultures provide an avenue to extract *candidate* CSC populations from individual (isogenic) MPE-tumors. This will allow us to test whether these cells can be discriminated in functional bioassays. Tumor heterogeneity in MPE-primary cultures is evidenced by variable immunolabeling, differences in colony-morphology, and differences in proliferation rates of cell subpopulations. Collectively, these data justify the ongoing development of the MPE-model for the investigation of intratumoral heterogeneity, tumor-TME interactions, and phenotypic validation of *candidate* lung CSC, in addition to providing direction for the pre-clinical development of rational therapeutics.

## Introduction

Lung cancer is the leading cause of cancer-mortality in the world. Current therapy is relatively ineffective, and the 5-yr survival rate is approximately 15%. Intratumoral heterogeneity possibly underlies resistance of lung cancers to current therapies; thus, accounting for intratumoral heterogeneity may be an important key to developing successful treatment strategies for lung cancer. Unfortunately, current models of lung cancer are limited in their scope to study tumor heterogeneity. Malignant pleural effusions (MPE) offer a unique opportunity to culture a wide variety of cancer cells from a single individual, in order to delineate and characterize the range of intratumoral heterogeneity found in advanced lung cancer.

The rationale for choosing the MPE, a regionally advanced stage of lung cancer that portends a poor prognosis, as a model to investigate intratumoral heterogeneity is twofold. First, an evolutionary model of carcinogenesis predicts that advanced disease states are more likely to depict heterogeneity [1]. Second, based on personal observations made over several years of studying MPE [2], [3], [4], [5], we knew that primary cultures derived from MPE initially displayed marked culture heterogeneity on their way to establishing morphologically homogeneous cancer cell lines. However, we had not previously investigated the biological or temporal basis of these observations in a prospective manner.

There are no established ways to culture MPE with the goal of maintaining intratumoral heterogeneity. Thus, we set out to develop a primary culture model *de novo*. This model incorporates the MPE-fluid component and extracted stromal cells in a tumor microenvironment (TME) that closely simulates the *in situ* milieu. Our data indicate that incorporation of these elements seems to preserve tumor heterogeneity. Since tumor heterogeneity may arise due to a hierarchical progression of transformed epithelium [6], [7], we hypothesized that included in the mix of tumor cells in MPE are cells which may function as cancer stem or progenitor cells. This manuscript provides proof of concept that *candidate* CSC can be fractionated from MPE primary cultures. This evidence justifies the further development of the MPE model for the examination of intratumoral heterogeneity and the study of *candidate* CSC-phenotypes.

## Materials and Methods

### MPE isolation, processing, and culture

**Figure 1:**
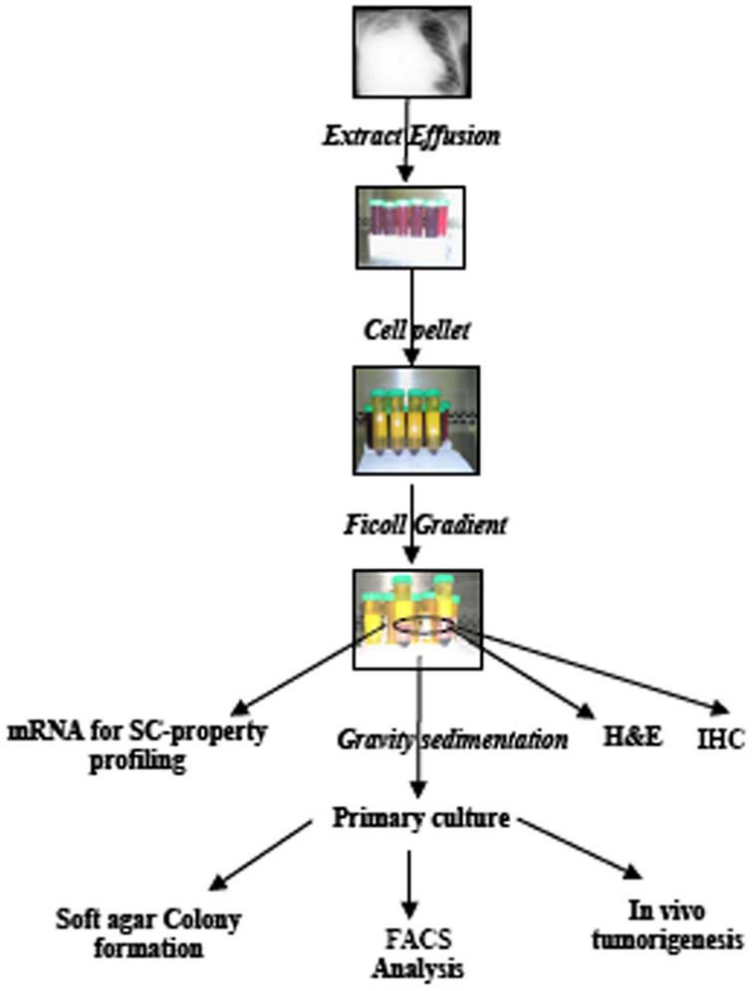
MPE-Fractionation strategy. MPE was extracted from the pleural cavity of lung cancer patients. The collected MPE was *sedimented (200×g)* and the *cell pellet* was separated from the MPE fluid. The cell pellet was resuspended in minimum essential medium containing MPE-fluid and layered on a *Ficoll gradient*. The nucleated cell fraction, which was largely devoid of erythrocytes in most cases, was collected from the interpose of Ficoll gradient and suspension medium, and processed for staining (*H&E, IHC*), *molecular analyses* and *primary culture*. The primary cultures are maintained in pcm, and are further analyzed for *soft agar colony formation*, *FACS analysis* and *in vivo tumorigenesis*.

All subjects underwent written informed consent by a process approved by the institutional review board at the Veterans Affairs-Greater Los Angeles Healthcare System (VAGLAHS). All subjects were veterans and active or former smokers, and MPE-specimens were acquired by large volume thoracenteses. Samples were processed as described in **Figure 1**. Briefly, following centrifugation (200×g, 20 minutes, room temperature), cell pellets were resuspended in a ficoll density gradient. The MPE-supernatant was sterile filtered and used for the formulation of the ***p***rimary ***c***ulture ***m***edium [pcm; DMEM-H (HyClone, UT) +30% v/v sterilely filtered MPE-fluid component+Penicillin-G/Streptomycin 1000 U/ml and Amphotericin B 0.25 µg/ml (Omega Scientific, CA)]. The selection of the 30% v/v fraction of the MPE-fluid component was empirically derived. Because the key soluble and/or cellular components which contribute to the maintenance of tumor heterogeneity in MPE-primary cultures is (are) not known, and since there is effusion-to-effusion variability in the concentrations of soluble factors, the selection of 30%v/v MPE-fluid component was based on observation of primary cultures by light microscopy. Briefly, we monitored three different MPEs in primary cultures containing either 100%, 70%, 50%, 30% and 10% MPE-fluid component. We evaluated culture integrity and variability by microscopy, and measured the fractions of floating dead cells (by trypan blue staining) in each condition over several days. There were no apparent qualitative differences in culture integrity or variability, and no quantitative differences in floating dead cells amongst the 70%, 50%, and 30% v/v MPE-fluid conditions. The 100% and 10% v/v MPE-conditions had an increase in cell death in two out of three effusions. This observation, combined with the practical consideration that MPE-fluid component was *the* limiting factor for duration of experimentation with primary cultures, led us to use the 30% v/v MPE in the remaining cases.

To establish primary cultures, the nucleated cell pellet was extracted from the ficoll gradient, washed with DMEM-H, and after aliquots were separated for storage, initial molecular analyses and cytopathology, several primary cultures were seeded. These were directly observed on a daily basis, and *pcm* was replaced at every 5–7 days. Kinetic growth analyses of primary cultures were performed on three distinct MPE specimens. For these determinations, primary cultures were seeded in parallel in 48 well plates (Corning Incorporated, NY), at a density of 2×10^4^ cells/well in 500 µl of culture media. To count, floating cells in suspension were collected first, after which the adherent populations were gently washed with phosphate buffered saline (PBS), and detached (Trypsin-EDTA, Sigma MO). The detached cells were then added to the initial cell suspension, and total live cells (Trypan blue dye exclusion) were manually counted by hematocytometer at designated time points. Such studies underlie the reported results that MPE-primary cultures grew at highly variable and slow growth rates.

### Antibodies

The following antibodies were used for immunohistochemistry (IHC) and/or flow cytometry (FACS): anti-CD 44 (Mouse monoclonal, Abcam # 16728 for IHC; Mouse Anti-Human IgG2b CD44-FITC, BD Biosciences # 555478 or PE- labeled mouse Anti-Human CD44, BD Pharmingen # 555479 for FACS), anti-uPAR (Mouse monoclonal, Santa Cruz Biotechnology # sc-13522), anti-ALDH1A1 (Rabbit monoclonal, Abcam # 52492), anti-CD166 –FITC (Mouse monoclonal; Abcam # 33403); primary unlabeled anti-cMET (mouse IgG2a, Abcam # 49210), primary unlabeled anti-MDR-1 (Mouse monoclonal, Chemicon # Mab4338), and anti-uPAR (Santa Cruz Biotech # 13522). Secondary antibodies used for the study: Goat F(ab')2 Anti-Mouse IgG (H+L)-PE-Cy.5.5 (Caltag laboratories # M35018) and Goat Anti-Mouse F(ab')2 IgM (Jackson ImmunoResearch).

### Cytopathology and Immunolabeling

Cell clusters were examined using phase contrast microscopy (Leica - Leitz DMRBE) on covered glass slides, or by light microscopy following fixation and staining. For the latter, cells fixed in ethanol (Fischer Scientific, PA) or Z-fix (Anatech, MI) were sedimented to generate a cell button, which was paraffin embedded. For IHC, sections (5 µm) were deparaffinized and rehydrated in xylene and ethanol. Antigen retrieval [10 mM citric acid (pH 6), 70°C, 30 min, twice with intervening water wash) was carried out, following which the slides were cooled to room temperature and sequentially rinsed with deionized water and PBS. Endogenous peroxidase activity was quenched (PBS +2% hydrogen peroxide), and the specimens were sequentially blocked [1% bovine serum albumin/PBS, room temperature 1 hr, followed by mouse serum (Santa Cruz Biotechnology) for 30 min, room temperature]. Tissue sections were then washed twice with PBS, incubated with the secondary antibody (HRP-conjugated Goat anti mouse, Santa cruz Biotechnology, 30 min, room temperature), rinsed with PBS and exposed to the DAB substrate kit (Vector Laboratories, Burlingame, CA). Negative controls typically utilized the secondary antibody alone in the absence of labeling with the primary. The stained sections were observed under the microscope (Leica - Leitz DMRBE or Olympus IX71) and the captured images were analyzed using the Openlab software. The positively stained cell areas were estimated using Image Pro Plus software. Cell clusters were manually defined using phase images and the irregular AOI tool and the resultant groups were segmented based on an empirically determined positive staining threshold. Percent of positively stained cells was estimated based on the fractional area of staining within the total cluster area.

### Reverse transcriptase-PCR (RT-PCR)

RNA was extracted from the primary MPE and culture isolates using Trizol reagent and Fast Track 2.0 mRNA isolation kit (Invitrogen Inc., Carlsbad, CA). 500 ng of the mRNA was reverse transcribed using the RT kit (Invitrogen, Inc., Carlsbad, CA) following the manufacturer's recommendations. Two µl aliquot of the synthesized cDNA was used for PCR for the amplification of PTEN, Oct4, Bmi1, hTERT, SUZ12 and EZH2 genes. The primers used were as follows: *PTEN* Forward - 5′ GGACGAACTGGTGTAATGATATG 3′, Reverse- 5′ TCTACTGTTTTTGTGAAGTACAGC 3′, *Oct4* Forward- 5′CAACTCCGATGGGGC CCT 3′, and Reverse -5′ CTTCAGGAGCTTGGCAAATTG 3′ *Bmi1* Forward - 5′ AATCTAAGGAGGAGGTGA 3′, Reverse- 5′ CAAACAAGAAGAGGTGGA 3′, *hTERT* Forward -5′ GGAATTCTGGAGCTGCTTGGGAACCA 3′, Reverse- 5′ CGTCTAGAGCCGGACACTCAGCCTTCA 3′, *SUZ12* Forward – 5′ GATAAAAACAGGCGCTTACAGCTT 3′, and Reverse 5′ – AGGTCCCTGAGAAAATGTTTCGA – 3′, *EZH2* Forward 5′ TTGTTGGCGGAAGCGTGTAAAATC 3′, Reverse 5′ TCCCTAGTCCCGCGCAATGAGC 3′. For *PTEN* amplification, the conditions were as follows: initial denaturation at 94°C for 4 minutes, followed by 32 cycles at 94°C for1 minute, 57.5°C for 1 minute, and 72°C for 3 min. A final extension at 72°C for 10 minutes was utilized. Amplification conditions for *Oct4* were an initial denaturation for 5 min at 95°C, followed by 32 cycles at 95°C for 30 seconds, 58°C for 30 seconds and 72°C for 30 seconds with a final extension at 72°C for 5 min. Amplification conditions for *Bmi1*: initial denaturation at 94°C for 4 min followed by 32 cycles at 94°C for 1 min, 53°C for 1 min, 72°C for 1 min, and a final extension for 7 min at 72°C. For *hTERT* the amplification conditions were: an initial denaturation at 94°C for 4 min, followed by 32 cycles at 94°C for 50 seconds, 52°C for 50 seconds, 72°C for 1 min, and a final extension at 72°C for 7 min. For SUZ12 and EZH2 amplification: initial denaturation at 94°C for 5 min, followed by 30 cycles of 94°C for 30seconds; 55°C for 30 seconds; 72°C for 30 s, and a 7 minute final extension at 72°C. PCR products were separated on 8% TBE (50 mM Tris borate pH 8.0, 1 mM EDTA) gels followed by Ethidium Bromide staining. Gels were analyzed using the Kodak 1D software.

### Flow cytometry and Aldefluor assay

FACS analyses of primary cultured MPE cells was performed by standard mutichannel FACS analysis using a FACSCalibur cytometer (Becton Dickinson, San Jose, CA) and FCS Express analysis software (De Novo Software, Ontario, Canada). Both non-adherent and adherent cells were collected and pooled. Cells in primary culture were detached using treatment with Trypsin/Versine, washed with PBS containing 2% BSA, and directly labeled with fluorochrome-tagged primary antibodies, or unlabeled primary antibody with fluorochrome-labeled secondary antibodies (as indicated below). Both primary and secondary antibody concentrations were consistently maintained at 1 µg/1×10^6^ cells; cells were labeled for 45 minutes at room temperature and sequentially washed three times in PBS containing 2% FBS, resuspended in PBS and maintained on ice before FACS analyses. The Aldefluor (Stemcell Technologies, Durham, NC, USA) assay, which measures aldehyde dehydrogenase (ALDH) activity, was performed according to manufacturer's guidelines. MPE-cells from primary culture were suspended in Aldefluor assay buffer containing the ALDH-substrate, Bodipy-aminoacetaldehyde (BAAA; 1.5 mM) and incubated for 50 min at room temperature. To verify specificity, a parallel specimen was incubated under identical conditions, but in the presence of a 10-fold molar excess of the ALDH-inhibitor, diethylaminobenzaldehyde (DEAB). This resultant decrease in the fluorescence intensity of ALDH-positive cells was used to compensate the flow cytometer analyses.

## Results

### MPE-characteristics, processing, and primary culture

To develop a model to study lung cancer endophenotypes in primary culture, we fractionated the cell and fluid compartments of MPE (**Figure 1**). The clinical characteristics of the effusions that were used to generate this dataset were typical of MPE (**Tables 1** **and** **2**, see below). Seven of nine effusions were malignant on the basis of cytopathological diagnosis (**Table 1**). In two MPE, the final cytopathology interpretation from 100 ml specimen sample was “highly suspicious but inconclusive”; these cases were included because growth of tumor was evident in primary cultures (including *in vivo* in one case, data not shown). Previous studies had long indicated that plating efficiencies and derivation of primary cultures from clinical lung cancer specimens is poor, and is dependent both on culture conditions and host-related factors [8], [9], [10], [11]. However, those studies did not supplement the primary tumor cultures with components from the *in situ* tumor microenvironment (TME), possibly because the primary or sole goal was to establish immortal tumor cell lines. In fact, in order to derive “pure tumor” cell lines in defined conditions, all earlier approaches took measures to minimize the “contamination” of cultures with TME-elements. Consequently, if distinct tumor cell subpopulations existed in individual tumors that depended on TME elements for survival, then using any artificial TME may have conferred a growth advantage to specific tumor cell subsets in a continuous culture system. Thus, it is possible that cancer cell models were “selected for” by the culture TME that was used to derive the tumor cell lines.

**Table 1:** Cytopathology of MPE and their in vitro growth.

Subject	Cytopathology	In vitro growth
106	NSCLC	ND
206	AdenoCa	ND
107	NSCLC	yes
207	Suspicious, NSCLC	yes
307	Large Cell Ca	yes
407	AdenoCa	yes
507	NSCLC	yes
607	Suspicious, SCCa	yes (primary passage path c/w SCCa)
707	Poorly differentiated SCCa.	yes

AdenoCa denotes lung adenocarcinoma, NSCLC denotes *N*on *S*mall *C*ell *L*ung *C*ancer (not specified), SCCa denotes lung squamous cell cancer, ND denotes not determined).

**Table 2:** Non Tumor Cellularity of the MPE.

MPE Cell Counts and Differentials	Mean±Standard Deviation (n = 9)	MPE in which wbc-diff is ≥1% total
RBC	158061±172443 rbc/µl	N/A
WBC	681±560 wbc/µl	9/9
Lymphocytes	60±26%	9/9
PMN	23±27%	8/9
Monocytes	3±3%	9/9
Macrophages	6±8%	8/9
Mesothelial Cells	4±4%	8/9
Other (eosinophils, plasma cells)	6±13%	4/9

Counts and differential were obtained by manual cytometer reading of Giemsa-Wright stained cytology slides in the VAGLAHS hematopathology laboratory. The data is cumulatively presented. The numbers in column two indicate the numbers and percentages of various cell types, and column three indicates the fraction of MPE in which the various cell types were identified. These data affirm that the cell counts of the effusions collected were typical of MPE.

We reasoned that the full complement of tumor cell endophenotypes would be best studied in primary cultures that incorporated autologous TME. Thus, in an attempt to maintain intratumoral heterogeneity *ex vivo*, both the tumor-accompanying nucleated cell population and the fluid component of MPE were used to enrich the primary culture-TME. To overcome the previously recognized inefficiency for establishing primary tumor cultures, and based on empirical observations described in the methods, we selected the “optimal” condition as being 30% MPE-fluid component admixed v/v into base medium containing antibiotics (**p**rimary **c**ulture **m**edium, or pcm). In *pcm*, both primary culture and serial passages were sustained with higher diversity in morphology, and cultures appeared to be more robust (as compared to parallel growth in fetal bovine serum, data not shown). Moreover, the utilization of a 30% v/v-fraction of MPE-fluid allowed us to conserve this limiting reagent for longer durations of experimentation with primary cultures. Using this methodology, we established primary cultures of MPE-tumors from all seven attempts.

Preliminary analyses indicated that the supernatant was highly enriched with inflammatory cytokines, with concentrations of interleukin (IL) 1, IL 6, C-X-C chemokine ligand 10 (IP10), C-C chemokine ligand 2 (MCP1), and vascular endothelial growth factor (VEGF) that were estimated to be >10 ng/ml. These and other factors, which likely originated from tumor, stromal and/or circulating cells in MPE (**Table 2**), contributed to a complex mix of cytokines, chemokines and growth factors in the MPE-fluid component. With respect to the cellular MPE-components, nucleated cell counts ranged from 1.3×10^8^ to 2.5×10^9^ cells per liter of effusion. The predominant circulating cell type in the TME were lymphocytes (mean±StDev: 60±26% out of total 681±560 wbc/µl), with significant (>1%) fractions of PMN, macrophages, monocytes and mesothelial cells (**Table 2**) in nearly all effusions. Thus, in terms of their circulating cell composition and biochemistry (they were all exudates) [12], the effusions we studied were typical of MPE.

### Evidence of intratumoral heterogeneity in extracted MPE-tumor specimens

**Figure 2:**
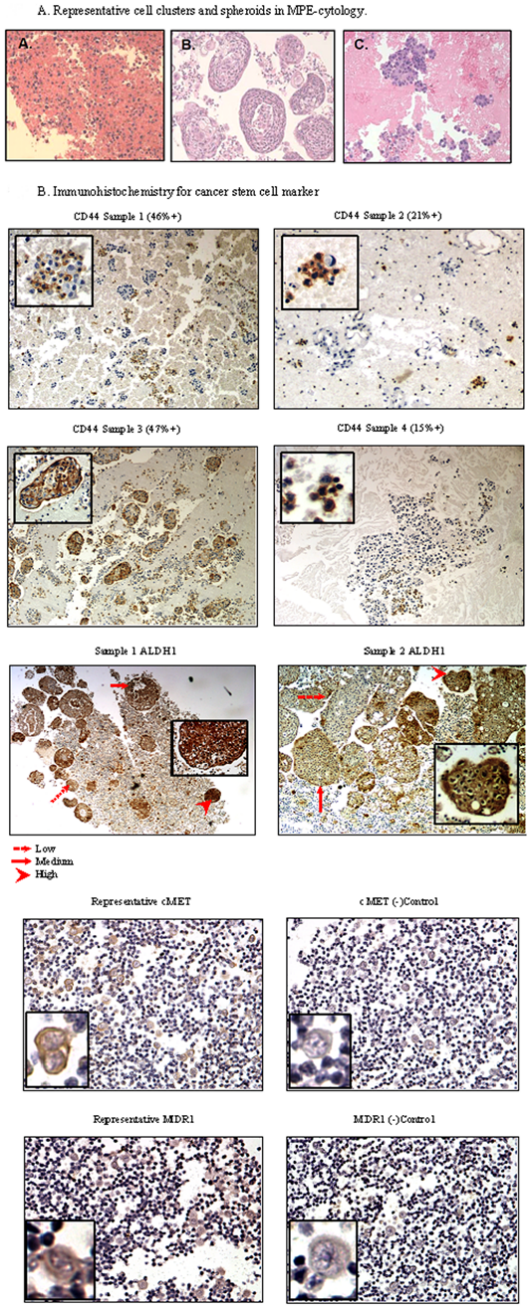
(A) Representative cell clusters and spheroids in MPE-cytopathology. Representative H& E of tumor specimens derived from MPE showing clusters and organized spheroids of varying morphology, and cell/stromal compositions (20×or 100×). (B) IHC for candidate CSC marker expression. Tumor specimens derived from MPE were immunolabelled for *candidate* CSC markers, including CD44 (40×and 400×), cMET (100×and 400×), MDR-1 (100×and 400×), and ALDH-1 (40×and 200×).

**Figure 2A** displays representative H&E stained cytopathology after ficoll density gradation. As shown, the MPE tumor was variably contained within indistinct clusters of cells of varying compositions, or as well organized spheroids. Closer examination suggested that both of these tumor cell conglomerates were themselves organized into distinct microdomains. We based this suspicion on the observation that when we labeled specimens for *candidate* CSC-markers, we often found staining within discrete foci in the aggregates (**Figure 2B**). For example, we stained MPE pathology specimens for the fractional expression of CD44. CD44, the cell surface receptor for hyaluronate [13], had been utilized as a surface label to select CSC. CD44 was previously used alone [14] and in conjunction with other cell surface markers to sort CSC from various epithelial malignancies, in both human and murine model systems [15], [16], [17], [18], [19], [20], [21]. All MPE specimens displayed a CD44+ fraction, which ranged from an estimated 8% to 47% of nucleated cells by immunohistochemistry (IHC) (**Figure 2B**, **Figures S1a and S1c**). In addition to CD44, cell fractions also displayed other *candidate* CSC-markers, cMET [22], [23] and MDR-1 [24], [25] (**Figure 2B**). Previously, other researchers had also exploited differences in xenobiotic metabolism of cells to segregate CSC from the tumor mix. One such technique utilized the Aldefluor™ assay (StemCell Technologies), which segregated *candidate* CSC on the basis of ALDH1A1 activity [26], [27], [28]. Like CD44, we found that cells that labeled for ALDH1 were seen in aggregated pockets within MPE-tumors (**Figure 2B**, **Figure S1b**). Marked variability of staining was evident even within an individual specimen. Thus, one could find discrete pockets of weak, medium and strong ALDH1 staining (**Figure 2B**). Foremost, these data indicated that individual MPE specimens had diverse immuno- and metabolic phenotypes. Moreover, if the *candidate* CD44 and ALDH-labels were valid surrogate markers for CSC, then CSC seemed to reside in discrete protected microdomains (niches) in the MPE-tumor clusters. Finally, although the temporal expression and functional correlation of these CSC-labels has yet to be determined, the data suggested that cells expressing *candidate* CSC markers could be potentially separated from the MPE-tumor mix.

### MPE-tumors express CSC-markers implicated in progenitor cell expansion or pluripotency programs

**Figure 3:**
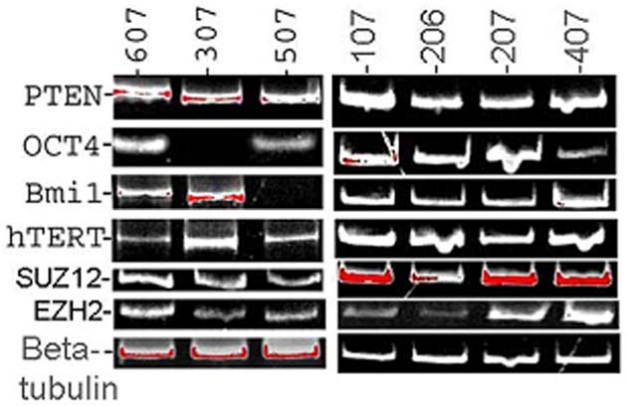
*MPE primary cultures express candidate CSC-molecular markers.* Expression profiles of PTEN, Oct4, hTERT, BMI1, SUZ12, and EZH2 were studied by reverse transcriptase-PCR. RNA extracted from the nucleated cell pellet of MPE was reverse transcribed and the cDNA was used in the PCR-amplification of the respective genes. PCR products were separated on 10% TBE gels, followed by ethidium bromide staining, and analyzed using the Kodak 1D software. PTEN, hTERT, SUZ12, EZH2 were uniformly expressed in all MPE, while BMI1 and Oct4 expression was not detected in individual samples. Beta tubulin was used as a loading control.

In general, tumors arise because of an abnormal arrest during tissue-differentiation [29], [30]. One of the first manifestations of the differentiation-arrest is that there is an expansion of the progenitor cell pool. Thus, molecular signatures of progenitor cells may serve as *candidate* CSC-biomarkers. In this regard, animal studies had implicated PTEN for the appropriate maintenance and differentiation of the peripheral lung progenitor cell population [31]. PTEN promoter silencing is evidenced in human lung cancer [32], implicating this pathway in its development and/or progression. Telomerase (hTERT) activation contributes to lung cancer pathogenesis [33], and hTERT is commonly activated in lung cancer. Along with p16 (INK4A), hTERT seems to be required for cell immortality that characterizes both stem cells and tumor cells [34], and its expression may indicate a dynamic change in the fraction of the immortalized phenotype [35], [36]. Shared markers between *candidate* CSC and stem cells also include pathways that mediate cell self-renewal and pluripotency. Oct4 is an embryonal marker that is associated with pluripotency, and is commonly used to label the CSC-phenotype [21], [37]. Similarly, the regulation of the pluripotent state is epigenetically controlled by structural changes in chromatin [38], [39], [40]. Transcriptional control during the pluripotent state is applied by the polycomb group (PcG) of proteins that work to modify chromatin structure. SUZ12, EZH2, and Bmi1 are components of PcG complexes, and because their expression in development is characteristic of tissue stem cells, they have also been used to label the *candidate* CSC-phenotype [6], [7], [41]. To test if these molecular signals of *candidate* CSC could be detected in MPE-tumors, RNA was extracted from the nucleated cell fractions and RT-PCR for these markers was performed. We found that these *candidate* CSC-biomarkers were expressed in different MPE-tumors (**Figure 3**). These data suggested that the lung CSC-phenotype was maintained in MPE despite the inflammatory milieu of the MPE-TME, contrary to some conventional postulates regarding the CSC niche environment. Perhaps, this was because the CSC were protected (as described) from the soluble TME in the tumor conglomerates that are evidenced in MPE. More importantly, these results also set the stage for dynamically tracking these molecular signals as experimental changes into the culture conditions are introduced in efforts to enrich for the CSC-phenotype.

### Evidence of intratumoral heterogeneity in MPE-primary cultures

**Figure 4:**
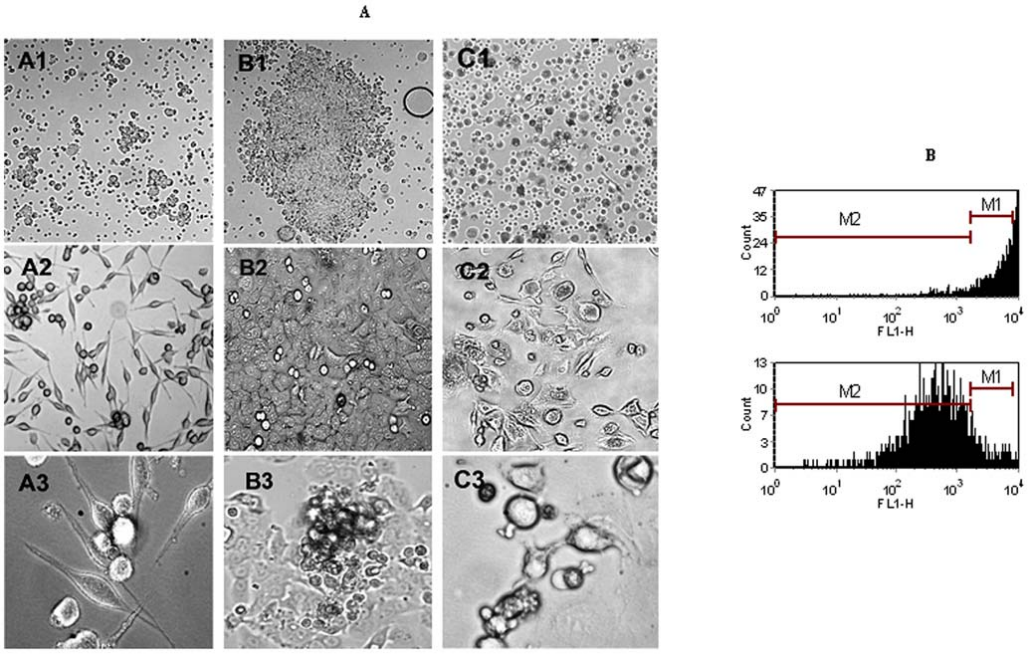
(A) Morphological heterogeneity in primary culture. Three different MPE (Sample A, Sample B and Sample C) cultured in pcm were evaluated for colony morphology. Each column (A, B, and C) represents a distinct specimen. Representative photomicrographs of colony heterogeneity by phase-contrast microscopy on days 3, 15 and 20 of primary culture (rows 1, 2 and 3, respectively) are presented. The photomicrographs in Rows 1, 2 and 3 are at magnifications of 40×, 100×and 400×, respectively. (B) MPE-subpopulations in primary culture can be fractionated on the basis of differences in proliferation: Representative flow histograms of Day 1 versus Day 9 of a CFSE-labeled primary culture. With expansion, subpopulations that are proliferative lose the CFSE label, and those which are quiescent retain the label. In this example, *on day 1, 96.14%* of the counts lie within the region gated by M1; on *day 9, 8.36%* of the counts lie within the region gated by M1.

MPE-primary cultures were established in pcm. In these conditions, the primary cultures displayed diverse morphologies (**Figure 4A**), and variably slow growth rates. The primary cultures typically took several weeks to “mature” (as defined by growth in the culture vessel approaching 70–80% confluence). Over this interval, the clusters and spheroidal structures that were observed in the initial MPE-cytopathology were not well conserved. Nevertheless, the primary cultures displayed a phenotypic heterogeneity that had not previously been examined. During their evolution, primary cultures were always comprised of colonies with varying morphologies (floating aggregates that exclude trypan blue, giant cell colonies, fibroblastoid and cobblestoned clusters), despite being in the same flask with an apparently identical TME (**Figure 4A**). These colonies seemed to expand at varying rates, suggesting that they had different proliferative indices, despite being in a “common” environment. We reasoned that if CSC, like tissue progenitor cells, had a reduced turnover, then differences in proliferation would allow us to segregate fractions enriched for CSC. To test whether such a strategy was feasible with MPE- primary cultures, we developed a live cell sorting strategy that used carboxyfluoroscein succinimidyl ester (CFSE) in a test-labeling scheme to fractionate cells based on different replication indices. CFSE is a membrane permeable reagent that is cleaved by intracellular esterases to yield a fluorescent amine-reactive metabolite which remains in the cytoplasm for weeks. Every time cells undergo division, the amount of CFSE present in each daughter cell is halved. Cells that are not replicating retain the label. Thus, *if* CSC are slowly replicating, *then* the label retaining population should be enriched for CSC. Although the cellular lineages that comprise the label retaining subset need to be better defined, these pilot, proof-of-concept studies suggested that MPE-tumor fractionation based on differences in proliferation indices was feasible (see the label-retaining cell population-**M1** that emerges over time in *pcm;* **Figure 4B**). In summary, differences in morphology, proliferation, surface marker and metabolic properties possibly reflect discrete endophenotypes of cancer cells in MPE. Although the functional correlates associated with each of these features have yet to be further explored, our results indicate that in MPE-primary cultures, the investigation of intratumoral heterogeneity is feasible.

### Rationally fractionating MPE-primary cultures to sort the putative CSC-subpopulation

**Figure 5:**
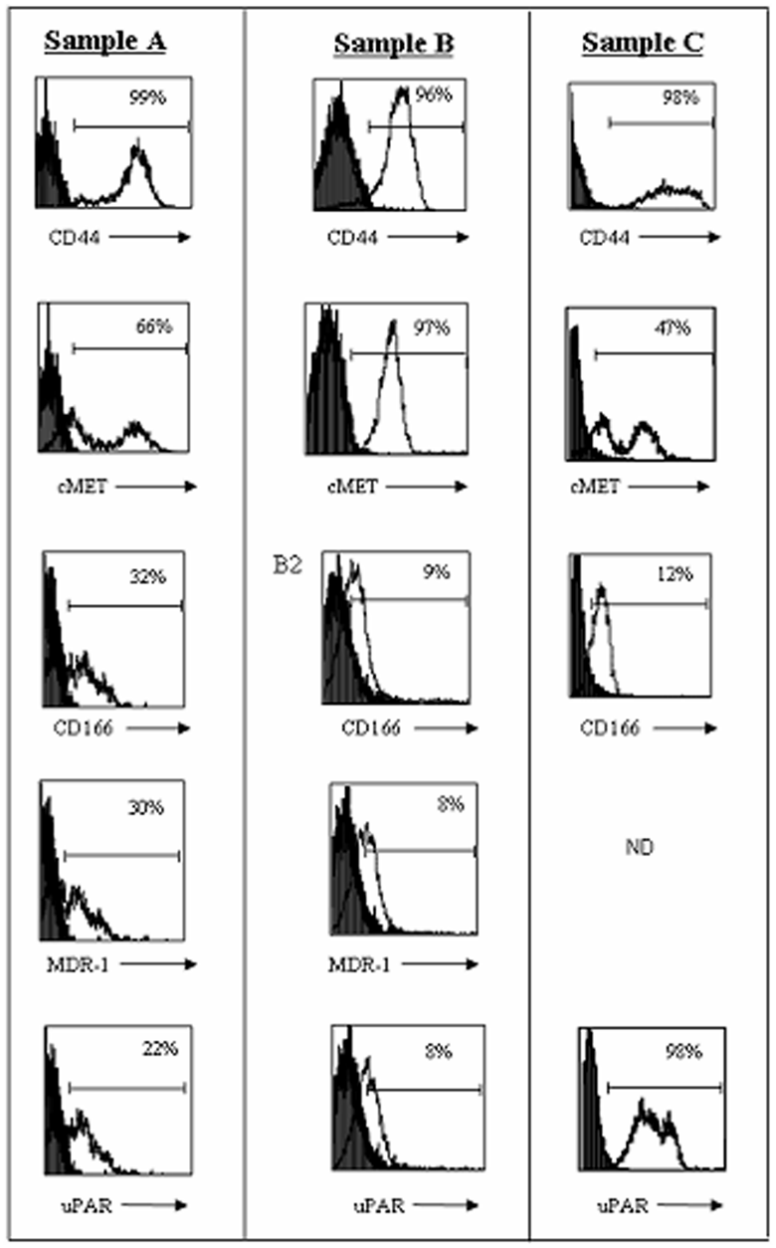
Fractional expression of candidate CSC-markers by FACS in MPE-primary cultures: Standard multi-channel FACS analyses of primary cultured MPE cells was performed using pooled MPE culture cells. Three different MPE (Sample A, Sample B and Sample C)-primary cultures in pcm were collected and labeled for *candidate* CSC-marker expression (CD44, cMET, CD166, MDR-1 and uPAR). Numbers at the upper right corners of each FACS-histogram represent the % of cells that are positive, as defined by cells displaying fluorescence exceeding the 95th percentile of cells stained with isotype matched control antibody.

**Figure 6:**
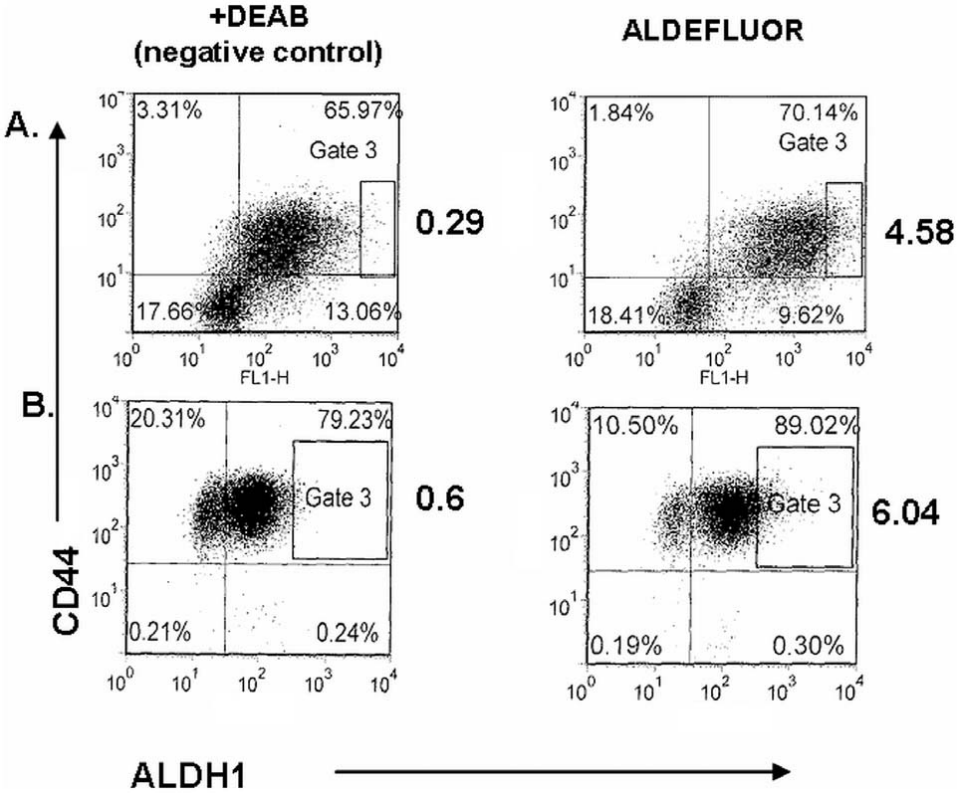
Aldefluor positive expression in MPE-primary cultures. Depicted are FACS-dot histograms of two distinct MPE-primary cultures. The intensity of Aldeflour expression (representing ALDH-activity) is on the abscissa; intensity of CD44 expression is on the ordinate. The dot plot figures on the left panel show control (ALDH-negative) cells in presence of ALDH inhibitor DEAB (+DEAB, negative control); ALDH-positive cells are shown in the right panel in Gate 3, in the absence of DEAB. Aldefluor+cells are depicted in gate 3; note that these cells also stain intensely for CD44.

With molecular signatures suggesting that CSC are present in MPE, we next tested if we could capture the *candidate* CSC from MPE-tumors. On maturation, MPE-primary cultures were sorted on the basis of *candidate* CSC-markers. Interestingly, in each case tested so far, the surface immunophenotype of cells in primary culture generally indicated an apparent expansion in the CD44+ fraction (**Figure 5**). Whereas cells were nearly uniformly positive for CD44 expression, fractions were more variably positive for cMET, CD166 [42], and uPAR [43] expression (**Figure 5**). Additionally, using a fluorescent assay that was reported to sort putative CSC from lung cancer, breast cancer, and multiple myeloma [26], [27], [28] on the basis of differential ALDH activity, a small fraction of the MPE-derived primary tumors displayed the ALDH^hi^/CD44^+^ phenotype (**Figure 6**). Collectively, these data indicated that *candidate* CSC-fractions could be segregated from primary cultures of MPE-tumor. In future studies, these fractions can now be compared to isogenic tumor cells for validation of the *candidate* “CSC-phenotypes” by bioassay.

## Discussion

Lung cancer is the leading global cause of cancer death in both men and women [44], but its molecular and cellular pathogenesis is not well understood [45]. Lung cancer diagnoses within the four major histological types are based on the preponderance of a specific histopathology by light microscopy. However, intratumoral heterogeneity has long been recognized to be a common occurrence in lung cancer [46], [47], [48], [49], [50]. This heterogeneity is evidenced throughout the course of the disease; cigarette smoke-exposed lung mucosa that appears morphologically normal displays diverse gene-expression profiles during pre-malignancy, and many/most lung cancers display mixed morphologies. Our overall hypothesis is that the observed differences in the molecular and morphological features of different tumor cells in individual lung cancers may have functional correlates.

Unfortunately, current models of lung cancer are limited in their scope to study tumor heterogeneity. Transgenic animal models are only provisional models for human disease for several reasons. For example, there are significant differences in the comparative anatomy and physiology between the mouse and human lung and there is a lack of certainty regarding the key gatekeeper mutations that result in the development of lung cancer in humans. Thus, the applicability of animal models to human disease remains imprecise. Similarly, current clinical research paradigms often survey genomic and gene expression profiles to catalog subtypes of lung cancer for diagnostic and prognostic purposes. However, data acquisition for genetic array analyses also overlooks tumor heterogeneity. Thus, even when methods for molecular profiling aim to collect data from “homogeneous” samples, inconsistencies in both the genomic and expression signatures remain evident. These inconsistencies likely reflect both inter- and intra-tumoral heterogeneity, and a new, complementary phenotype-based approach to stratify and prognosticate lung cancer should be considered. Here, we have provided proof of concept that MPE-primary cultures may be used to investigate intratumoral heterogeneity and to isolate *candidate* lung CSC. In this respect, our results not only advance the notion that there is diversity, but that this diversity can now be studied in culture, using methods we have described in this report.

The mechanisms underlying the development of tumor heterogeneity are unclear. Perhaps, this heterogeneity is attributable to an evolutionary process [1], and/or it may be the culmination of hierarchical progression of disease [6], [7]. Either way, our MPE-model offers a unique opportunity to culture a wide variety of cancer cells from a single individual, and to experimentally determine the molecular basis for an observed phenotype. For example, proponents of the cancer stem cell (CSC)-hypothesis argue that rare cancer cells bearing stem cell traits can be isolated from advanced tumors, and that these cells can recapitulate the full heterogeneity evidenced in the parental tumor in implanted xenografts [14], [15], [18], [27], [28], [42], [51], [52], [53], [54], [55], [56]. CSC may also be endowed with programs that form the basis for cytotoxic drug resistance [57] and tumor invasion. However, it is also recognized that in advanced malignancies, “undifferentiated” cancer cells emerge, which often display epithelial to mesenchymal transition (EMT) and are associated with the acquisition of invasive traits [19], [41]. Importantly, the undifferentiated cells have overlapping features with the stem cell phenotype. For lung cancer, it is not known whether the undifferentiated cells bearing stem cell features in advanced malignancy are in fact *bona fide* CSC. However, to answer this important question, the MPE-model is an appropriate, clinically relevant prototype to determine if cells that are segregated on the basis of *candidate* CSC markers are predictive of a distinct tumorigenic or invasive phenotype.

To ascertain the role of CSC in lung cancer pathogenesis, several groups have embarked on independent efforts to isolate and characterize *candidate* lung CSC [24], [42], [58], [59], [60]. Each investigator has employed different tactics and models to characterize lung CSC. Our efforts are unique in that we utilize *clinical* MPE specimens, and establish primary culture in an autologous culture TME. It remains to be seen whether such differences in techniques or sources of tumor will translate into differences in the cancer endophenotypes that are selected, or differences in the biological profiles of the *candidate* CSC which emerge from these efforts. Our approach clearly poses several important challenges. The kinetics of primary cultures are very variable, and importantly, colony-growth *within* an individual culture is heterogeneous. Observations of primary cultures enable us to envision how the process of establishing model immortalized cell lines may select the most resilient tumor cell subpopulations in a given culture environment over time, while leaving a fraction or major proportion of cells extinct. If that is the process by which cell lines are developed, then the contributions of the extinct subpopulations would be largely unaccounted for in cell line models. Based on our early observations, we postulate that our model enables the prolonged maintenance of some tumor cell subpopulations that would have died out in other conditions. Although the key soluble and/or cellular components which contribute to the tumor heterogeneity and/or maintenance of the *candidate* CSC in MPE-primary cultures have yet to be defined, it is important to note that cells bearing surrogate labels for cancer stem cells are included in the MPE-tumor mix.

However, we also note that our primary cultures evolve in terms of their structure and cellular compositions as they expand *in vitro*. The markers and labels that are used to identify and extract CSC also display dynamic changes. Based on our observations, if these *candidate* labels are valid surrogates for the CSC-phenotype, then we cannot be confident that this is a “rare” population. One possibility for the observed changes is that they represent the transition between the *in situ* to the *in vitro* state. As described, the well organized 3-dimensional tumor spheroids/clusters that are extracted from the patient are not well preserved in primary culture. It is possible that as the relatively organized structures disintegrate *in vitro*, the cancer cells within are exposed to soluble factors that are typically excluded from the extracellular matrix in the organized structures *in situ*. Perhaps, upon being exposed to novel factors and cytokines in the MPE-fluid microenvironment, the tumor cells are induced to undergo epithelial to mesenchymal transition (or a transition to the *candidate* CSC-phenotype). Either way, whether the *candidate* CSC markers can be specifically correlated with a distinctive phenotype needs to be experimentally determined, and our MPE-culture model will allow us to make these determinations.

At this juncture, our studies are unable to provide definitive proof that a discrete subpopulation of tumor cells within the MPE is capable of more efficient tumorigenesis than isogenic counterparts in an *in vivo* model. Although not described earlier in this report, the prospective experimental design and model (subcutaneous implantation of tumor in *scid* mice) we utilized for a phenotypic readout did not yield tumors with high efficiency from primary MPE isolates, and did not enable us to establish a reliable baseline for the cell numbers needed for *in vivo* tumorigenesis. Although one can invoke many reasons for why the phenotypic outcome measure we chose failed, the exercise was empirically informative by suggesting that a new transplantation model will likely need to be developed. Thus, in future studies that are undertaken to compare engraftment efficiencies of *candidate* CSC versus isogenic control tumor cells by limiting dilution analyses, we have proposed to develop a new animal transplantation model. Given that a recent report suggested effective transplantation of TME components along with tumor cells in a transplantable animal model of lung cancer [61], we envision that this model may prove useful for our purpose.

Importantly, in an effort to develop alternative phenotypic outcome measures to select the *candidate* CSC-phenotype, we were able to establish cultures *in vitro* with high efficiency (7/7 attempts), using the novel strategy that utilized an autologous tumor microenvironment. In this primary culture model, we have been able to provide a proof-of-concept that ***1)*** *candidate* lung CSC are present in this milieu, ***2)*** *candidate* lung CSC can be maintained over time in this primary culture environment, and ***3)*** that we can live sort *candidate* lung CSC from these primary cultures to evaluate their phenotype in various bioassays. These new developments set the stage for experimentation along pathways that are distinct from *in vivo* tumorigenesis by limiting dilution analyses. For example, we can now propose to test whether MPE-tumors that are segregated on the basis of *candidate* CSC-markers will display differences in *in vitro* surrogate measures of the CSC-phenotype (soft-agar colony formation, drug resistance, and/or matrigel invasion) from isogenic tumor cells that don't express *candidate* CSC markers. In summary, our results argue for the ongoing development of the MPE-primary culture model, and set the stage for correlating observed phenotypic differences with distinctive molecular signatures. Our hope is that by characterizing the molecular basis for specific tumor endophenotypes in MPE, we will be able to better design rational therapeutic combinations that are more predictive of clinical efficacy.

## Supporting Information

Figure S1 Figure S1a: Representative images of CD44 staining with negative controls. Figure S1b: Representative images of ALDH staining with negative control. Figure S1c: Additional representative images depicting CD44 staining within microdomains of MPE-tumor clusters.
(7.73 MB TIF)
