# Supplementary figures and images for: The Malignant Pleural Effusion as a Model to Investigate Intratumoral Heterogeneity in Lung Cancer
Source: PLoS One. 2009 Jun 12;4(6):e5884. doi: 10.1371/journal.pone.0005884 (PMC2697051; doi:10.1371/journal.pone.0005884)

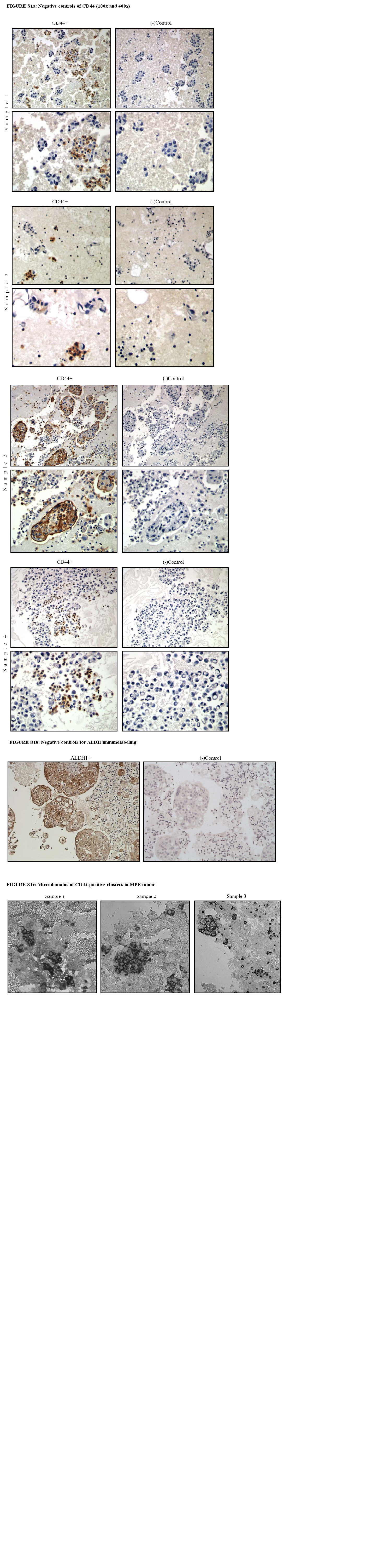

Supplement: Figure S1 — a: Representative images of CD44 staining with negative controls. Figure S1b: Representative images of ALDH staining with negative control. Figure S1c: Additional representative images depicting CD44 staining within microdomains of MPE-tumor clusters. (7.73 MB TIF) [file pone.0005884.s001.tif]
